# The Association of Smartphone Usage with Sleep Disturbances among Medical Students

**DOI:** 10.1055/s-0044-1788772

**Published:** 2024-09-25

**Authors:** Mohammed Alhafi, Rashed Matrood, Mohammad Alamoudi, Yazzed Alshaalan, Mohammed Alassafi, Aamir Omair, AbeerAl Harthi, Laila Layqah, Mutaz Althobaiti, Jinan Shamou, Salim Alawi Baharoon

**Affiliations:** 1 College of Medicine, King Saud bin Abdulaziz University for Health Sciences, Riyadh, Saudi Arabia; 2 King Abdullah International Medical Research Center, Riyadh, Saudi Arabia; 3 Ministry of National Guard-Health Affairs, Riyadh, Saudi Arabia; 4 Department of Pediatrics, King Abdullah Specialized Children Hospital, Riyadh, Saudi Arabia; 5 Department of Medicine, King Abdul-Aziz Medical City, Riyadh, Saudi Arabia; 6 King Saud bin Abdul-Aziz University for Health Sciences, Riyadh, Saudi Arabia

**Keywords:** medical students, sleep, smartphones, Pittsburgh Sleep Quality Index, smartphone addiction scale short version

## Abstract

**Background** Smartphones have become an important and vital instrument that all medical students utilize, but the usage of such devices has been found to be connected to sleep disturbances. The aim of this study was to estimate the prevalence and the relationship between smartphone addiction and poor sleep quality among medical students.

**Methods** A cross-sectional study was conducted among the fifth- and sixth-year medical students at King Saud bin Abdulaziz University for Health Sciences in Riyadh, Saudi Arabia. A self-administered questionnaire was distributed to investigate the relation between smartphone usage and sleep disturbances. The questionnaires included demographic details, Pittsburgh Sleep Quality Index (PSQI) and smartphone addiction scale short version (SAS-SV).

**Results** All 251 respondents had smartphones that were utilized for social media, communication, studying, etc. Most of the students were identified as smartphone addicts (65%), and 75% of the students had poor sleep quality. Sleep quality was found to be poor in 145 (85%) students with smartphone addiction. Smartphone addiction was significantly associated with poor sleep quality (odds ratio [OR]: 4.271; 95% confidence interval [CI]: 2.300–7.933; *p* < 0.001). Gender and academic year were not significant predictors of poor sleep quality with *p* -values of 0.668 and 0.361, respectively. Smartphone addiction was significantly more prevalent among female students (80%) compared to male students (60%; *p* = 0.004), with the mean addiction score of 43.5 ± 11.5 and 33.5 ± 9.1, respectively ( *p* < 0.001).

**Conclusion** Our study shows a significant association between smartphone addiction and poor sleep quality. It is strongly recommended that counseling services be provided to medical students to assist those suffering from smartphone addiction and sleep difficulties.

## Introduction

A smartphone “is a mobile phone that performs many of the functions of a computer, typically having a touchscreen interface, internet access, and an operating system capable of running downloaded apps.” 1 Phones in the past were utilized only for communication, but nowadays, phones are considered a mobile computer that provide its user with a connection to the internet, ability to play multimedia content, and many other benefits. These benefits have made smartphones part of people's lives to the point that some become addicted to it.

In 2016, a study about the relationship between sleep disturbances and mobile phone usage was conducted on 203 undergraduate medical students from India, using the Pittsburgh Sleep Quality Index (PSQI), and it was found that up to 61% of the students used smartphones at nighttime and 72% of them were having poor sleep quality. 2 Another study conducted at Guru Gobind Singh Medical College in India in 2015 on 1,000 medical students showed that 76% of the participants had a smartphone and nighttime usage of phones was highly associated with difficulty in waking up, inactivity, low focus, and missed classes. 3

A study on sleep disturbance patterns conducted in Riyadh, Saudi Arabia, on male medical students at Imam Mohammed University using a self-administered questionnaire concluded that the majority of the students experience sleep disturbances with different patterns. 4 Another study was done to understand sleep deprivation impact on different students' abilities and skills at King Saud University, Riyadh, which revealed a sleep deprivation prevalence of 32% among male students and 29% among female students. The results also show that not getting adequate sleep time because of long studying hours was the medical students' chief issue regarding sleep. 5

The aim of this study was to estimate the proportion of medical students in King Saud bin Abdulaziz University for Health Sciences (KSAU-HS) who use smartphones and suffer from sleep disturbances and stress. It also aimed to estimate the hours of usage, evaluate the sleep patterns, and identify the possible consequences of sleep disturbances among the medical students on different aspects of life.

## Methods

A cross-sectional study was conducted among medical students at KSAU-HS at Riyadh, Saudi Arabia, from January to March 2020. A self-administered questionnaire was used to assess sleep pattern and smartphone usage. The study population comprised 377 fifth- and sixth-year students (both male and female) selected using the nonprobability convenience sampling technique. Data on demographics, sleep pattern, and smartphone usage were collected. The PSQI was used to assess sleep quality. It is a self-rated questionnaire used to assess the pattern and quality of sleep to differentiate and distinguish good and poor sleep. It is composed of 19 items with a Likert scale and 7 scoring components that measure subjective sleep quality over the last month. Finally, it gives a global score that is indicative of poor sleep if it is ≥5. 6 7 8 The smartphone addiction scale short version (SAS-SV) was used to assess the smartphone addiction among KSAU-HS medical students. It is composed of 10 items along with a Likert scale that results in a total score that ranges between 10 and 60, with a cutoff point of 31 for males and 33 for females indicative of smartphone addiction. 7

Statistical software IBM-SPSS version 24 was used for data analysis. The descriptive statistics were presented as percentage and frequency for categorical variables, while the mean and standard deviation were presented for the numerical variables. The chi-squared test was used for comparing the association between the categorical variables. Independent sample *t* -test was used to compare the mean scores among categorical variables, and logistic regression analysis was used to evaluate the predictive value of several factors on the outcome. The level of significance in this study was set at a *p* -value of less than 0.05.

## Results

A total of 350 questionnaires were distributed (90 females and 260 males), of which 251 (71.7%) questionnaires were returned. The response rate for male and female students were 72.6 and 68%, respectively, and was similar between the fifth- and sixth-year students. Study participants ranged in age from 20 to 30 years, with a mean age of 21.9 ± 1.65 years, and 189 (75%) of them were males. The respondents were divided equally between the fifth and sixth-year students (50% each; Table 1 ).

**Table 1:** Baseline characteristics and difference between participants with good and poor sleep quality

Parameter	Good sleep quality 62 (24.7%)	Poor sleep quality 189 (75.3%)	Total 251 (100%)	*p* -value
**Age (y)**	21.73 ± 1.4	22.05 ± 1.72	21.97 ± 1.65	0.177
**Gender**				
Male	48 (19.12%)	141 (56.1%)	189 (75.2%)	0.782
Female	14 (5.57%)	48 (19.12%)	62 (24.7%)	
**Academic year**				
Fifth	34 (13.5)	92 (36.65%)	126 (50.1%)	0.487
Sixth	28 (11.15%)	97 (38.64%)	125 (49.8%)	
**SAS-SV category**				
Addict	25 (9.96%)	139 (55.37%)	164 (65.3%)	<0.001
Nonaddict	37 (14.74%)	50 (19.9%)	87 (34.6%)	
SAS-SV score	29.9 ± 10.46	37.97 ± 10	35.98 ± 10.6	<0.001
PSQI score	3.016 ± 1.0	8.07 ± 2.6	6.82 ± 3.2	<0.001

Abbreviations: PSQI, Pittsburgh Sleep Quality Index; SAS-SV, and smartphone addiction scale short version (SAS-SV)

Based on SAS-SV, smartphone addiction was positive in 164 (65.3%) students. There was a significant difference between smartphone addiction and gender. Sixty percent of male students were addicted to smartphone compared to 80% of female students ( *p* = 0.004) with the mean addiction score being significantly higher in female students (43.5 ± 11.5 vs. 33.5 ± 9.1, *p* < 0.001; Table 2 ). More male students were present in the high-risk and no-addiction score zones compared to female students; however, the number of students in these zones in both groups was very low. There was no significant difference between the academic year and smartphone addiction ( *p* = 0.85). The prevalence of poor sleep quality according to the PSQI score among students was 189 (75.3%) with a mean score of 6.8 ± 3.2. There was no significant difference between gender and poor sleep quality; however, the mean PSQI score was significantly higher among female students (7.56 ± 3.7 vs. 6.58 ± 3, *p* = 0.038; Tables 1 and 2 ). We found a significant association between smartphone addiction and poor sleep quality and PSQI score ( *p* < 0.001; Table 1 ). Logistic regression analysis revealed that smartphones addicts were 4.2 times more likely to experience poor sleep quality (odds ratio [OR]: 4.271; 95% confidence interval [CI]: 2.300–7.933, *p* < 0.001). Gender and academic year were not significant predictors of poor sleep quality, with *p* -values of 0.668 and 0.361, respectively ( Table 3 ).

**Table 2:** Comparison between mean PSQI and SAS-SV scores among genders

Parameter	Male	Female	Total	*p* -value
PSQI score	6.58 ± 3.0	7.56 ± 3.7	6.82 ± 3.2	0.038
SAS-SV score	33.5 ± 9.1	43.5 ± 11.5	35.9 ± 10.6	<0.001
**SAS-SV category**				
Addict	114 (60.3%)	50 (80.6%)	164 (65.3%)	0.004
Nonaddict	75 (39.7%)	12 (19.4%)	87 (34.7%)	

Abbreviations: PSQI, Pittsburgh Sleep Quality Index; SAS-SV, and smartphone addiction scale short version (SAS-SV)

**Table 3:** Logistic regression analysis comparing gender, academic year, and SAS-SV result on sleep quality

Variable	*B*	S.E.	Wald	df	Sig.	Exp(B)	95% CI
Gender (1 = male)	–0.160	0.373	0.184	1	0.668	0.852	0.411–7.933
Academic year	0.282	0.309	0.834	1	0.361	1.326	0.724–2.430
SAS-SV (1 = addict)	1.452	0.316	21.122	1	<0.001	4.271	2.300–7.933

Abbreviations: CI, confidence interval; SAS-SV, smartphone addiction scale short version; S.E., standard error.

Looking at smartphone addiction, our study showed that 56 (22%) students agreed that smartphone usage had caused them to miss work and 58 (23%) stated that they have trouble concentrating in the class, at work, or on assignments. Moreover, 69 (28%) agreed that they cannot live without a smartphone. In addition, 77 (31%) participants use their smartphones for longer than they anticipated. Even though it has a significant impact on their daily life, 47 (19%) participants would never stop using their smartphones ( Table 4 ).

**Table 4:** Students response on smartphone addiction scale short version (SAS-SV) statements

Question	Strongly disagree, *N* (%)	Disagree, *N* (%)	Weakly disagree, *N* (%)	Weakly agree, *N* (%)	Agree, *N* (%)	Strongly agree, *N* (%)
Missing planned work due to smartphone use	30 (12)	31 (12.3)	26 (10.4)	56 (22.3)	52 (20.7)	56 (22.3)
Having a hard time concentrating in class, while doing assignments, or while working due to smartphone use	19 (7.6)	32 (12.7)	32 (12.7)	58 (23)	70 (27.9)	40 (15.9)
Feeling pain in the wrists or at the back of the neck while using a smartphone	49 (19.5)	60 (23.9)	25 (10)	47 (18.7)	43 (17)	27 (10.8)
Won't be able to stand not having a smartphone	23 (9.2)	31 (12.4)	29 (11.6)	44 (17.5)	69 (27.5)	55 (21.9)
Feeling impatient and fretful when I am not holding my smartphone	37 (14.7)	61 (24.3)	38 (15)	47 (18.7)	34 (13.5)	34 (13.5)
Having my smartphone in my mind even when I am not using it	54 (21.5)	64 (25.5)	46 (18.3)	40 (15.9)	27 (10.8)	20 (8)
I will never give up using my smartphone even when my daily life is already greatly affected by it	31 (12.4)	53 (21)	45 (17.9)	47 (18.7)	40 (15.9)	35 (13.9)
Constantly checking my smartphone so as not to miss conversations between other people on Twitter or Facebook	22 (8.8)	46 (18.3)	40 (15.9)	64 (25.5)	44 (17.5)	35 (13.9)
Using my smartphone longer than I had intended	12 (4.8)	17 (6.8)	25 (10)	52 (20.7)	68 (27)	77 (30.7)
The people around me tell me that I use my smartphone too much	60 (23.9)	68 (27)	41 (16.3)	34 (13.5)	25 (10)	23 (9.2)

Regarding sleep habits, around 25% of the students go to bed around 12 a.m. and 40% of the students wake up by 7 a.m. The average time spent in bed before falling asleep was 30 minutes. The average number of hours of actual sleep was 6.48 ± 1.39, while the average hours in bed was 7.03 ± 1.34 hours. The minimum hours of sleep were 3 hours, and the maximum was 10 hours.

## Discussion

Our study shows that 85% of senior year medical students have a significant association between poor sleep and smartphone addiction. Poor sleep quality was present in 75.3% of the surveyed students, 77% among female students and 74% among male students. Because the PSQI is only intended to screen for bad sleep and not to diagnose it, a diagnostic interview should be conducted to validate any diagnosis of poor sleep.

The mean PSQI score among the group defined as having a poor-quality sleep was significantly higher in the female students but was not different between the fifth- and sixth-year students. This may reflect a true gender difference or alternatively a biased finding because the total number of female students in the surveyed group were less than male students.

It appears that Saudi Arabian medical school students frequently get poor-quality sleep. In one study with a mean PSQI score of 7.11, around 76% of the medical students examined in Riyadh had poor sleep quality. 9 Comparably, between 64 and 75% of medical students at various Saudi universities in various areas had poor sleep quality. 10 11 12 13 14 15 Not only do medical students frequently have poor sleep, but residency training is also associated with poor sleep. 16 Problems with sleep quality do not seem to be exclusive to medical felids; rather, Saudis seem to experience them frequently. According to one study, one in three Saudi individuals suffer from inadequate sleep each night. 17

Regarding smartphone addiction, a study conducted in 2018 on smartphone addiction among students in Al-Imam Mohammed bin Abdul Aziz University using SAS indicated that 65% of the study samples were within the addicted category, which is similar to our findings (65%). 18 Another study in 2016 that used a different assessment tool than SAS in King Saud University found that the smartphone addiction percentage among students was 48%. 19 The smartphone addiction percentage in this study was higher in comparison with global results using the same assessment tool (SAS-SV). A study done in 2015 among medical students in India found that smartphone addiction was 34%. 3 In 2017, a study in South Korea reported that 30% of medical students were smartphone addicts. 20 In a recent published systemic review analyzing more than 30,000 participants in 20 studies, smartphone addiction was found in around 29%, which is still lower than our findings. 21 This higher prevalence of smartphone addiction among students from different universities in the kingdom of Saudi Arabia is alarming and concerning. In our study, smartphone addiction was also significantly higher among female students; there are mixed results in this regard in the existing literature. In the above-mentioned systemic review, smartphone addiction was more prevalent among male students compared to female students. 21 Another study done in the kingdom of Saudi Arabia showed no significant difference between smartphone addiction and gender, 22 and other studies support our findings. 23 24 These differences might be explained by the different addiction scales used or a true variation due to demographic or socioeconomic factors. In our study, this higher prevalence of addiction among female medical students might be due to the smaller sample size of females; however, such a finding, if further consolidated by a larger-scale study, might warrant more exploration of different demographic and socioeconomic backgrounds, and pattern of smartphone usage.

This study concluded that a significant association between sleep quality and smartphone addiction was present among medical students. To the best of our knowledge, only one study discussed the association between phone usage and sleep quality in Saudi Arabia. It was done in 2018 in King Abdulaziz University and concluded that increased usage of mobile phones had a negative effect on sleep. 25 More studies on this association worldwide support our findings. 2 3 26 27

## Conclusion

This study reveals that excessive use of smartphones is highly prevalent among medical students and is linked to poor sleep quality. It is our recommendation that medical institutions focus on the importance of the availability of counseling services to medical students and create health education programs that could help those with smartphone addiction and sleep disturbances. Furthermore, students' lifestyle can be changed positively, which would improve their quality of life and their productivity.
